# UV-C Radiation as a Factor Reducing Microbiological Contamination of Fish Meal

**DOI:** 10.1155/2014/928094

**Published:** 2014-01-21

**Authors:** Krzysztof Skowron, Justyna Bauza-Kaszewska, Zbigniew Dobrzański, Zbigniew Paluszak, Karolina Jadwiga Skowron

**Affiliations:** ^1^Department of Microbiology, Faculty of Pharmacy, Nicolaus Copernicus University, Collegium Medicum of L. Rydygier, 9 M. Skłodowskiej-Curie Street, 85-094 Bydgoszcz, Poland; ^2^Department of Microbiology and Food Technology, Faculty of Agriculture and Biotechnology, University of Technology and Life Sciences, 6-8 Bernardyńska Street, 85-029 Bydgoszcz, Poland; ^3^Department of Environment Hygiene and Animal Welfare,The Faculty of Biology and Animal Science, Wroclaw University of Environmental and Life Sciences, 38C Chełmońskiego Street, 51-630 Wrocław, Poland

## Abstract

Fish meals, added to feeds as a source of protein, may contain pathogenic bacteria. Therefore, effective methods for their sanitizing, such as UV-C radiation, are needed to minimize the epidemiological risk. The objective of this study was to evaluate the effect of UV-C radiation on the sanitary state of fish meals. The research materials included salmon and cod meals. Samples of the fish meals were inoculated with suspensions of *Salmonella*, *E. coli*, enterococci, and *C. sporogenes *spores and exposed to the following surface UV-C fluencies: 0–400 J*·*m^−2^ for bacteria and 0–5000 J*·*m^−2^ for spores. For the vegetative forms, the highest theoretical lethal UV-C dose, ranging from 670.99 to 688.36 J*·*m^−2^ depending on the meal type, was determined for *Salmonella*. The lowest UV-C fluency of 363.34–363.95 J*·*m^−2^ was needed for the inactivation of *Enterococcus *spp. Spores were considerably more resistant, and the UV-C doses necessary for inactivation were 159571.1 J*·*m^−2^ in salmon meal and 66836.9 J*·*m^−2^ in cod meal. The application of UV-C radiation for the sanitization of fish meals proved to be a relatively effective method for vegetative forms of bacteria but was practically ineffective for spores.

## 1. Introduction

Fish meal production is of great importance for the efficient management of organic matter. This industrial process makes some amounts of wastes of animal origin available for further sustainable use and also provides high-protein compounds to produce feeds for farm animals, mostly swine and poultry [1].

According to the regulation (EC) no. 1774/2002 of the European Parliament and of the Council, a fish meal is processed animal protein obtained from marine animals, with the exception of marine mammals. The technology of processing this protein, also strictly determined by the current regulations, demands that the final product is microbiologically safe. However, some authors explicitly report meat bone and fish meals added to feeds as a source of a high incidence of human and animal diseases. This resulted from the presence of *Salmonella *in those meals [2–4]. Therefore, attempts were made towards minimizing the degree of epidemiological threat. However, further detection of bacterial contaminations resulted in the conclusion that a secondary contamination of this material had to be considered, which occurred probably during transport or processing.

The literature data on the methods used to decontaminate fish meals are relatively rare. Malicki et al. [5, 6] investigated the effect of short-chain organic acids and pressurization on the inactivation of *E. coli* and *Salmonella* in fish meal samples. Both treatments resulted in satisfactory level of enteric pathogens reduction (4-5 log cfu·g^−1^) and the authors suggested their application for decreasing the microbiological risk related to contaminated fish meals.

One of the methods that can effectively reduce the number of undesirable microorganisms in a given environment is UV-C irradiation. The wavelength ranges between 200 and 280 nm, and the highest bactericidal effectiveness is obtained by radiation at 254 nm. Among the benefits resulting from the application of UV-C for decontamination, the most essential are the simplicity of the method, low dependency on pH and temperature, the lack of any toxic chemical substances applied, and the lack of byproducts and remains generated in the case of chemical reactions [7, 8]. The limited penetration ability of UV-C radiation is one of its disadvantages [9].

The aim of this study was to evaluate the effect of different doses of UV-C radiation on the sanitary state of fish meals. The efficiency of the studied methods was evaluated based on the elimination rate of *Salmonella*, *E. coli*, enterococci, and spores of *Clostridium sporogenes* introduced into the tested material.

## 2. Material and Methods

### 2.1. Research Material

The materials for the study included samples of two kinds of fish meal—salmon and cod. Cod meal contained on average 19.6% of total protein and 0.5% of crude fat. The content of Ca was 22–27% and that of P 13-14%. Salmon meal contained on average 27.4% of crude protein and 1.8% of crude fat. The content of Ca was 16–25% that of P 11-12%.

Production technology of these fish meals is the subject of a patent application at the UP RP (no. P-403123).

The research material was initially examined for the total number of microorganisms, moulds, and *Actinobacteria*, as well as for *Salmonella*, *E. coli*, enterococci, and spores of sulfite-reducing anaerobic bacteria.

### 2.2. Preparation of Bacteria and Spore Suspensions

Bacterial suspensions were prepared in sterile Ringer's solution based on 24-hour cultures of the tested bacteria on nutritional agar. The density of each suspension was estimated using a densitometer at 0.3 McF.

To obtain spores, 0.5 mL of *Clostridium sporogenes *culture in BHI broth stored at 4°C was introduced into 10 mL of liquid FTG medium and incubated under anaerobic conditions (Anaerobic System, Oxoid) for 24 hours at 37°C. Then, 0.5 mL of the culture was transferred from the FTG medium to 10 mL of Duncan-Strong medium and incubated under anaerobic conditions for 7 days at 37°C. Then, the inoculated Duncan-Strong medium was centrifuged at 3000 rpm for 15 minutes, washed twice and suspended in 50 mL of sterile distilled water, in order to obtain a suspension with a density of 10^7^ cfu·mL^−1^.

### 2.3. Sample Inoculation

Samples of both kinds of fish meal were inoculated with suspensions of *Salmonella*, *E. coli*, bacteria of the genus *Enterococcus*, and spores of *Clostridium sporogenes* IW1306 (PKM Wrocław).

The 1 mL of each bacterial suspension and of the spore suspension was added to analytical samples of both types of fish meals with a weight of 7 g each and then stirred to obtain a “dough” consistency. The inoculated samples were dried for 45 min at 37°C.

### 2.4. Determination of UV-C Efficiency

Dried samples of both types were divided into 7 portions of 1 g each and spread as a thin layer on ceramic trays with an area of 6.25 cm^2^. The following fluencies of surface UV-C radiation were applied: 0, 10, 25, 50, 75, 100, and 400 J·m^−2^ for bacteria and 0, 100, 400, 1000, 2500, and 5000 J·m^−2^ for spores. The trays were placed below a UV-C radiator at a distance of 1 m, so that the cosine angle between the normal to the surface and the vector directed to the UV source was 0.

A radiator with a power of 30 W was applied for irradiation and the effective intensity of UV-C radiation at a distance of 1 m from the UV source was 2.3 W·m^−2^. Considering the location of trays with samples, the actual efficiency of UV-C radiation was calculated in line with the equation:

(1) $$\begin{array}{c} E=\frac{I}{r^{2}}\cos\alpha , \end{array}$$

where *E* is the actual intensity of radiation (W·m^−2^), *I* is the nominal effective intensity of radiation (W·m^−2^), *r* is the distance of the sample from the UV source (m), and *α* is the angle between the normal to the surface and the vector directed to the UV source, and in the experiment it accounted for 2.3 W·m^−2^.

The values of surface fluencies of a dose were obtained by selecting appropriate exposition times, which were calculated according to the equation

(2) $$\begin{array}{c} t=\frac{P}{E}, \end{array}$$

where *t* is the time of exposition (sec.), *P* is the surface fluency of dose (J·m^−2^), and *E* is the actual intensity of radiation (W·m^−2^).

The times needed to obtain the intended UV-C dose fluency are presented in Table 1.

### 2.5. Determination of the Number of Bacteria and Spores

The probable number of all of the studied bacteria was determined with the MPN method in a 3-tube set, making tenfold dilutions of the radiated samples in appropriate media. For the isolation of rod-shaped bacteria of the genus *Salmonella* 1% buffered peptonic water was used for initial multiplication (24 h at 37°C). Selective multiplication was carried out using the liquid medium according to Rappaport (24 h at 43°C). For culture on a solid medium, the selective BPLS agar medium was used (24 h at 37°C), onto which *Salmonella* grows in the form of pale-pink colonies, coloring the medium pink. Final identification involved using diagnostic sera according to the White-Kauffmann-Le Minor classification scheme.

The number of *E*.* coli* rods was determined with a series of tenfold dilutions in MacConkey's broth. The inoculated broth was incubated at 43°C for 24 h. The change of medium colour from purple to yellow and the presence of gas in the Durham tube were rated as a positive result. After incubation in broth, the material was cultivated on solid ENDO agar as selective medium. Plates were incubated at 43°C for 24 hours. Typical growth of *E. coli* rods on ENDO agar appeared in the form of dark red colonies with a green-gold fuchsine sheen. For final identification the API 20E test was applied.

Enterococci determination was performed using a broth with glucose and azide (48 h at 37°C) and agar with kanamycin, esculin, and azide (48 h at 37°C). Turbidity of the liquid medium with glucose and azide indicated the presence of enterococci in the sample. On the solid media with kanamycin, the growth of small colonies and a dark colour of the medium indicated the presence of enterococci. Final identification was performed using the serological Phadabac Strep Test.

In order to determine the number of *Clostridium sporogenes* spores in the studied material, a series of 10-fold dilutions was prepared in tubes containing 9 mL of peptonic water each. Then, to inactivate vegetative forms, the tubes were placed in a water bath at 80°C for 15 min. After cooling the tubes with dilutions, the number of spores was determined based on the results of cultures made with the deep method. For this purpose, 1 mL probes were taken from each dilution in 3 replicates and introduced into sterile Petri dishes and DRCM medium solidified with agar was poured over the probes. The cultures were incubated for 3 days at 37°C under anaerobic conditions. After the incubation, characteristic black colonies of *Clostridium sporogenes* were counted on each plate.

### 2.6. Statistical Analyses

The experiment was conducted in three replications. Based on the results obtained, theoretical lethal doses, inactivation rates, and doses causing a 90% reduction in the number of bacteria were calculated. The determined parameters were analysed statistically using the SAS 9.2. PL software, which allowed the identification of significant differences at the *P* ≤ 0.05 and *P* ≤ 0.01 probability levels by Tukey's tests.

## 3. Results

The initial investigations did not prove the presence of the chosen indicator bacteria or that of further fungi or *Actinobacteria *in the samples. The total number of microorganisms did not exceed 10^1^ cfu·g^−1^.

The present study proved a gradual elimination of the studied bacteria in both types of fish meal along with an increase in the surface fluency of the UV-C radiation dose (Tables 2–4). The initial concentration of microorganisms in the studied material each time was on a level of 10^5^ MPN·g^−1^ (Tables 2–4) and 10^6^ MPN·g^−1^ in the case of enterococci in the cod meal (Table 4). The highest applied dose of UV-C of 400 J·m^−2^ caused a 3-log reduction in the number of *Salmonella *(Table 2), more than a 4-log reduction in *E. coli* (Table 3), and the complete inactivation of enterococci (Table 4).

In case of spores of *Clostridium sporogenes*, however, changes in number under the influence of different UV-C fluencies were very small. The number of spores isolated from the studied meals remained on a level of 10^6^ cfu·g^−1^ (Table 5).

**Figure 1:**
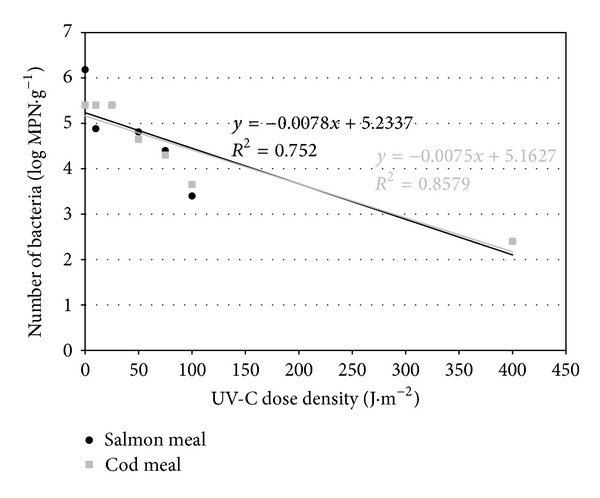
Regression lines describing inactivation kinetics of *Salmonella* spp.

**Figure 2:**
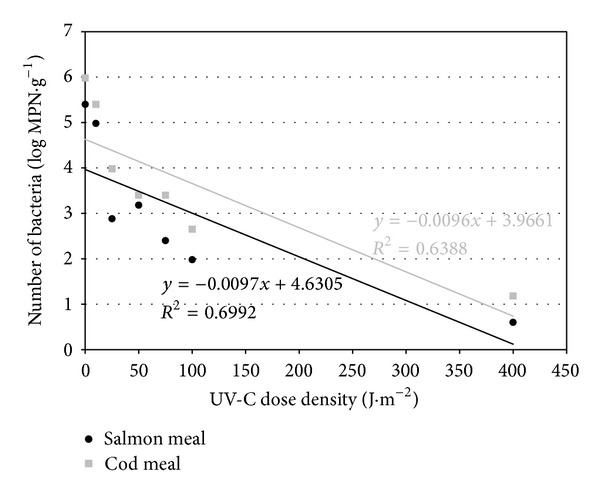
Regression lines describing inactivation kinetics of *E. coli*.

**Figure 3:**
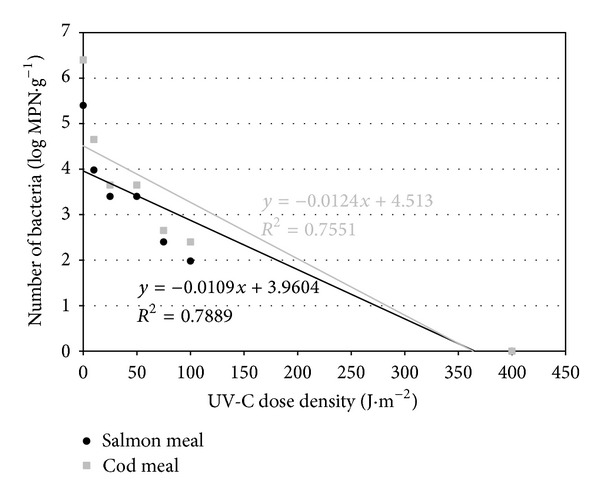
Regression lines describing inactivation kinetics of *Enterococcus *spp.

**Figure 4:**
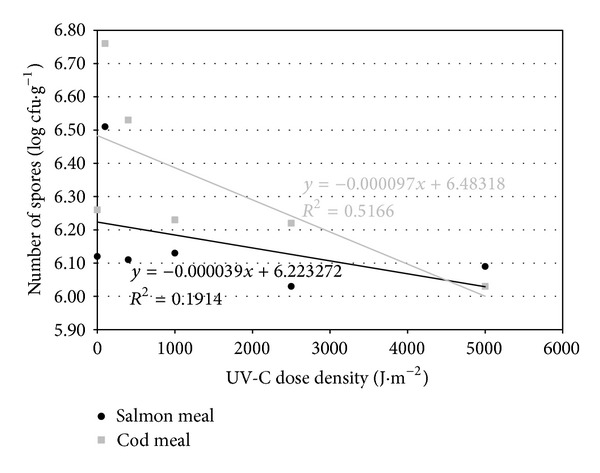
Regression lines describing inactivation kinetics of *Clostridium sporogenes* spores.

The theoretical lethal dose of UV-C radiation for *Salmonella*, calculated from the regression line (Figures 1, 2, and 3), was slightly higher for cod meal than for salmon meal (Table 6). The reverse tendency was observed for *E. coli *(Table 6), whereas in the case of enterococci, the theoretical lethal doses were practically identical for both types of fish meal (Table 6). The differences observed in theoretical lethal UV-C doses were not statistically significant (*P* > 0.05) (Table 6). In the case of *Clostridium sporogenes *spores, the theoretical UV-C dose fluency needed for their inactivation calculated on the basis of the regression equations (Figure 4) was significantly higher for the salmon meal than for cod meal (Table 6).

Highly statistically significant differences (*P* ≤ 0.01) in the resistance of the studied species of microorganisms to the inactivation by UV-C radiation were obtained. For the vegetative forms, the highest theoretical lethal dose, depending on meal type, was determined for *Salmonella*, ranging from 670.99 to 688.36 J·m^−2^ (Table 6). By contrast, the lowest UV-C dose (363.34–363.95 J·m^−2^) was needed for the complete inactivation of bacteria of the genus *Enterococcus* (Table 6). Spores appeared to be considerably more resistant and UV-C doses amounted to 159571.1 J·m^−2^ in the salmon meal and 66836.9 J·m^−2^ in the cod meal. These values were distinctly and significantly higher than those for vegetative forms of the studied bacteria (Table 6).

The calculated inactivation rate of *Salmonella *of 0.008 log MPN × (J·m^−2^)^−1^ was the lowest for the vegetative bacterial forms in both types of fish meals (Table 6). The highest inactivation rate, however, was observed in the case of enterococci: 0.011 log MPN ×  (J·m^−2^)^−1^ in the salmon meal and 0.012 log MPN × (J·m^−2^)^−1^ in the cod meal (Table 6). Statistically significant differences (*P* ≤ 0.05) were observed only between the inactivation rate of enterococci in the cod meal and *Salmonella *spp. in both types of fish meals (Table 6). *Clostridium sporogenes *spores, in turn, underwent a highly statistically significant slower elimination when compared with vegetative bacterial forms. In addition, the difference in inactivation rates depended on the type of fish meal (Table 6).

Based on regression analyses (Figures 1–4), UV-C radiation doses were calculated that caused a reduction in the number of the studied bacteria by 90% (*D*_90_) (Table 6). A decrease in the number of *Salmonella *and *E. coli *by 90% occurred at a higher dose in cod meal, while in case of enterococci it took place in salmon meal (Table 6). The observed differences connected with the meal type were not statistically significant (*P* > 0.05), with the exception of bacteria of the genus *Enterococcus *(*P* ≤ 0.05) (Table 6).

The highest *D*_90_ doses to obtain a 90% reduction of population number were observed in *Salmonella *(128.21–133.33 J·m^−2^), whereas the lowest doses (80.65–91.74 J·m^−2^) were found in enterococci (Table 6). The differences between the studied microorganisms were statistically significant (*P* ≤ 0.05) (Table 6).

In case of spores of *Clostridium sporogenes *the *D*_90_ UV-C doses were significantly higher (10309.3–25 641.0 J·m^−2^) than those determined for vegetative forms of the studied bacteria and the difference between the types of fish meal was also highly statistically significant (Table 6).

## 4. Discussion

The bactericidal effect of UV radiation is most of all due to the photochemical destruction of nucleic acids, resulting in the inhibition of cell reproduction. This effect is commonly applied in many branches of industry and economy. Special equipment has been developed for the effective disinfection of air, water, and sewage that is based on the emission of UV of appropriate wavelength [10, 11]. There are also many reports confirming its possible application, for example, in food industry to reduce the number of microorganisms developing on the surfaces of fruits, vegetables, eggs, or meat. This refers to both pathogens and saprophytes, the latter being harmless from the epidemiological point of view, but responsible for the microbiological decomposition of products [8, 10, 12].

Wong et al. [13] analysed the efficiency of inactivation by UV radiation in the cases of *Salmonella* Senftenberg and *Escherichia coli *inoculated on the surface of fresh pork meat and pig leather. The obtained results proved that, irrespective of the applied dose of radiation, *Salmonella *was characterized by a higher susceptibility to UV radiation than *E. coli*. Similar relationships were observed, when indicator bacteria were exposed to irradiation in sewage [14]. The applied dose of 100 J·m^−2^ caused a reduction in the number of *S. Enteritidis* by more than 2 log and *Enterococcus faecalis* by more than 1 log, whereas the number of *E. coli *decreased only by about 0.5 log.

The results of the current experiments indicated that, among the vegetative forms, *Salmonella *was characterized by the highest resistance to the effect of UV-C radiation (Table 6). Most susceptible were bacteria of the genus *Enterococcus, *for which a dose of 400 J·m^−2^ resulted in complete inactivation (Table 4).

de Nardi et al. [11] also confirmed that the UV radiation dose needed for the complete inactivation of indicator microorganisms is higher for *Salmonella *than for *E. coli. *Elimination of these bacteria from sewage required radiation doses of 320 J·m^−2^ and 110 J·m^−2^, respectively. Caretti and Lubello [15], in turn, reported that in sewage irradiated with UV *E. coli *survived considerably longer than enterococci. A similar susceptibility was observed in many studies concerning the effect of UV-C radiation on microorganisms of faecal origin [8, 16].

Differences in the obtained results may be determined by many factors, resulting both from the specificity of the environment of the microorganisms exposed to the UV radiation and from the properties of the studied strains. In liquid material, such as water, sewage, or food products intended for drinking, an enormous impact can be attributed to the thickness of the irradiated liquid and its turbidity and chemical properties, as well as the ability of microorganisms to form aggregates with molecules suspended in those liquids. These structures may exhibit protective functions against the applied irradiation [17, 18]. The study of Geveke et al. [19] indicates that penetration depth of 254 nm UV in liquid whole egg and liquid egg white was 0.066 mm and 0.085 mm, respectively. Guerrero-Beltrán and Barbosa-Cánovas [20] stated that the penetration of UV light into juices is about 1 mm. In the case of the typically superficial applications of UV, many authors stress the importance of factors such as the chemical composition of the irradiated substance and also the colour and topography of the surfaces, where the microorganisms are exposed to radiation [21]. Wong et al. [13] claimed that the uneven and porous structure of pork meat may hinder penetration of UV rays and results in a lower reduction in colonies of *Salmonella* Senftenberg than in the case of smooth surface such as leather. 

There are few sources concerning the penetration depth of UVC radiation into material with constant consistency. According to Manzocco et al. [22], the depth of UV-C radiation into the surface of fresh-cut apple was 0.20 mm. Since the thickness of meal layer in the present study was about 1-2 mm, a relatively high degree of reduction of vegetative forms of the studied microorganisms can be regarded as satisfactory.

In comparison with other types of radiation also used in decontamination processes, the ability of UVC radiation to penetrate both liquid and solid material is very low. The depth of penetration of an electron beam in most food stuffs is 5 cm, and gamma rays can penetrate rock to 30 cm and food to 40 cm. Higher ability to penetrate is reflected in the hygienization effectiveness of methods based on the effect of such radiation types. Gamma radiation in range from 3 to 20 kGy is applied for reduction of microbial population in dry food ingredients [23]. The gamma radiation dose needed to inactivate 1 log_10_ of *Salmonella* spp. and *E. coli* O157:H7 inoculated onto RTE (ready-to-eat) foods product was 0.61 and 0.36 kGy, respectively [24]. Similarly to the results of our investigation, the gamma radiation resistance of *E. coli* was lower than that reported for *Salmonella*.

The developmental stage of microorganisms subjected to inactivation is also of essential importance for the effectiveness of most sanitizing technologies. In this study spores of *Clostridium sporogenes *showed a considerably higher resistance to UV-C radiation than *Salmonella*, *E. coli*, or enterococci (Table 6). Even the use of very high doses did not reduce the number of spores considerably, which constantly remained at a level of 10^6^ cfu·g^−1^ (Table 5).

Only limited information about the effect of UV-C on bacterial spores in food products and animal feeds is available in literature. Chang et al. [16] report that spores present in sewage exposed to UV irradiation showed a 9 times higher resistance than bacterial cells in the vegetative stage. Nerandzic and Donskey [25] documented the efficiency of UV radiation in reducing the number of spores of *C. difficile *on various surfaces in hospital rooms. The authors addressed the phenomenon from an application's point of view, that is, enhancing the sporicidal efficiency of UV radiation by previous stimulation of germination of mature spores.

Susceptibility to UV radiation can also depend on the origin of spores. Li et al. [18] observed that *Bacillus subtilis* pores obtained from laboratory cultures were distinctly less resistant to UV rays than those isolated directly from the natural environment. A dose of 400 J·m^−2^  caused a decrease in their number by 1.5 and 2.5 log, respectively. Among other factors, which may influence the efficiency of spore inactivation, the authors mentioned their initial concentration and identified the bacteria species level. The latter factor, in turn, determines the specific spore structure and decides, for instance, how deep UV radiation can penetrate into the spore and what are the capacities of the species to repair UV damage.

Investigations carried out on the molecular level proved that the degree of spore dehydration and the structure of peptidoglycan in the cortex, which largely determine the thermoresistance, play a relatively small role in the induction of spore resistance to UV irradiation. In this process, the essential importance is attributed to the saturation of spore DNA with SASP—small acid-soluble proteins *α*/*β*-type [26–28].

More profound knowledge about the mechanisms involved in the bactericidal effect of UV radiation, connected with its inactivation efficiency, proved empirically in various environments, encourage to broaden the range of applications of this agent. The obtained results of the study may make the grounds for the initial evaluation of fish meal decontamination parameters. Their possible application would require considering many factors that may modify the course of decontamination process, such as the particle size of the given part of material, the thickness of layer subjected to radiation, or its color, taking into consideration the fact that other potential methods for inactivation of pathogenic microorganisms in meals, for example, involving exposing to the effect of gamma radiation or high temperature, in spite of proved effectiveness, need much higher financial outlays and taking additional precautions. However the further action towards the use of UV-C radiation to sanitize fish meals seems to be justified.

The results of the current study show that UV-C radiation can be an effective agent reducing the risk of spreading vegetative forms of pathogenic microorganisms that cause secondary contaminations of fish meals. This is of particular importance in view of the frequently signalling threats related to a possible contamination of fish meal products with *Salmonella*—the direct cause of dangerous infections in humans and animals.

The UV-C fluency that caused the complete inactivation of the studied bacteria should amount to about 700 J·m^−2^. Some differences between bacterial species were observed with respect to their resistance to UV-C radiation. The type of fish meal did not considerably affect the survival of vegetative forms of bacteria. However, some differences in inactivation rate were observed, depending on the fish meal type, especially in case of *Clostridium* spores. Because the treatment conditions (UV radiation source, temperature, and thickness of the radiated meal layer) were equal for both—salmon and cod meal—the most probable factor responsible for different inactivation rate was chemical composition and physical properties of the investigated material. Another possible cause may be some differences in the initial concentration of bacteria and spores in both types of fish meal.

Unfortunately, this study documented also a low efficiency of UV-C radiation in the reduction of *Clostridium* spores number. Current methods used for bacterial spores elimination are mostly based on their thermal inactivation. High efficiency of these technologies, however, is usually related to high costs and complicated equipment, necessary for their application. Moreover, heat treatment may result in irreversible alteration of biological and physicochemical features of the material subjected to the action of high temperature. The alternative, nonthermal methods of bacterial spores inactivation investigated in many scientific centers, take into consideration high pressure, different types of irradiation or chemicals as sporicidal agents. Stewart et al. [29] observed 2.5 log_10_ reduction of *Clostridium sporogenes* PA 3679 spores after pressurization treatment of 404 MPa at 25°C, pH 6, for 30 minutes. Lowering pH, lengthening the exposure time, raising the temperature, and adding sucrose laurate or nisin resulted in significantly increased effectiveness of HPP treatment. Inactivation of *Bacillus* spores, ranging from 2 to 5 log cycles, was achieved after irradiation with accelerated electrons (7.6 kGy) [30]. Studies of Lee and Pascall [31] demonstrated 5 log reduction of *C. sporogenes* spores viability after combined action of acidification and mild temperature. According to the presented results, all of investigated agents applied alone or combined with each other guarantee satisfactory level of bacterial spores elimination.

## Figures and Tables

**Table 1:** Exposition times of inoculated samples of fish meal needed to obtain the intended UV-C dose in case of a distance of 1 m between UV source and probe.

Time of exposition	Dose (J·m^−2^)
0 s	0
4 s	10
11 s	25
22 s	50
33 s	75
43 s	100
2 min 28 s	400
7 min 15 s	1000
18 min 7 s	2500
36 min 14 s	5000

**Table 2:** Changes in the number of *Salmonella* depending on the surface density of the UV-C radiation dose.

UV-C dose (J·m^−2^)	Number of *Salmonella* rods (MPN·g^−1^)	
	Type of fish meal	
	Salmon	Cod
Control	15.0 × 10^5^	2.5 × 10^5^
10	7.5 × 10^4^	2.5 × 10^5^
25	2.5 × 10^5^	2.5 × 10^5^
50	6.5 × 10^4^	4.5 × 10^4^
75	2.5 × 10^4^	20.0 × 10^3^
100	2.5 × 10^3^	4.5 × 10^3^
400	2.5 × 10^2^	2.5 × 10^2^

**Table 3:** Changes in the number of *E. coli *depending on the surface density of the UV-C radiation dose.

UV-C dose (J·m^−2^)	Number of *E. coli *(MPN·g^−1^)	
	Type of fish meal	
	Salmon	Cod
Control	2.5 × 10^5^	9.5 × 10^5^
10	9.5 × 10^4^	2.5 × 10^5^
25	7.5 × 10^2^	9.5 × 10^3^
50	15.0 × 10^2^	2.5 × 10^3^
75	2.5 × 10^2^	2.5 × 10^3^
100	9.5 × 10^1^	4.5 × 10^2^
400	0.4 × 10^1^	1.5 × 10^1^

**Table 4:** Changes in the number of *Enterococcus* spp. depending on the surface density of the UV-C radiation dose.

UV-C dose (J·m^−2^)	Number of enterococci (MPN·g^−1^)	
	Type of fish meal	
	Salmon	Cod
Control	2.5 × 10^5^	2.5 × 10^6^
10	9.5 × 10^3^	4.5 × 10^4^
25	2.5 × 10^3^	4.5 × 10^3^
50	2.5 × 10^3^	4.5 × 10^3^
75	2.5 × 10^2^	4.5 × 10^2^
100	9.5 × 10^1^	2.5 × 10^2^
400	n.d.*	n.d.

*n.d.: not detected.

**Table 5:** Changes in the number of *Clostridium sporogenes* spores depending on the surface density of the UV-C radiation dose.

UV-C dose (J·m^−2^)	Number of *C. sporogenes *spores (cfu·g^−1^)	
	Type of fish meal	
	Salmon	Cod
Control	1.32 × 10^6^	1.83 × 10^6^
100	3.23 × 10^6^	5.80 × 10^6^
400	1.30 × 10^6^	3.37 × 10^6^
1000	1.34 × 10^6^	1.70 × 10^6^
2500	1.08 × 10^6^	1.67 × 10^6^
5000	1.22 × 10^6^	1.07 × 10^6^

**Table 6:** Parameters describing inactivation kinetics of the studied bacteria in fish meal as affected by UV-C radiation.

Type of meal	Bacteria	Theoretical lethal dose (J·m^−2^)	Inactivation rate (log⁡MPN×(J·m^−1^))	*D*_90_ (J·m^−2^)
Salmon	*Salmonella *spp.	670.99^A,a^ (*±184.12*)*	0.008^A,a^ (*±0.0002*)*	128.21^A,a^ (*±23.17*)*
	*E. coli *	477.37^B,b^ (*±97.41*)*	0.010^A,a,b^ (*±0.0007*)*	103.09^A,b^ (*±31.67*)*
	*Enterococcus *spp.	363.34^C,c^ (*±102.02*)*	0.011^A,a,b^ (*±0.001*)*	91.74^A,c^ (*±14.08*)*
	Spores of *C. sporogenes *	159571.1^D,d^ (*±10593.3*)*	0.000039^B,c^ (*±0.000002*)*	25641.0^B,e^ (*±1006.3*)*

Cod	*Salmonella *spp.	688.36^A,a^ (*±113.25*)*	0.008^A,a^ (*±0.0006*)*	133.33^A,a^ (*±40.52*)*
	*E. coli *	413.14^B,b^ (*±127.55*)*	0.010^A,a,b^ (*±0.002*)*	104.17^A,b^ (*±17.94*)*
	*Enterococcus *spp.	363.95^C,c^ (*±92.09*)*	0.012^A,b^ (*±0.004*)*	80.65^A,d^ (*±10.55*)*
	Spores of *C. sporogenes *	66836.9^E,e^ (*±8369.12*)*	0.000097^C,d^ (*±0.000011*)*	10309.3^C,f^ (*±2603.6*)*

A, B, C,…: highly statistically significant differences at *P* ≤ 0.01.

a, b, c,…: statistically significant differences at *P* ≤ 0.05.

*Standard deviation.
